# The Gut-Brain Axis and the Microbiome: Clues to Pathophysiology and Opportunities for Novel Management Strategies in Irritable Bowel Syndrome (IBS)

**DOI:** 10.3390/jcm7010006

**Published:** 2018-01-03

**Authors:** Eamonn M.M. Quigley

**Affiliations:** Lynda K and David M Underwood Center for Digestive Disorders, Division of Gastroenterology and Hepatology, The Methodist Hospital, Houston, TX 77030, USA; equigley@houstonmethodist.org; Tel.: +1-(713)-441-0853

**Keywords:** irritable bowel syndrome, gut-brain axis, microbiota, microbiome, microbiota-gut-brain axis, antibiotics, probiotics

## Abstract

Irritable bowel syndrome (IBS) is one of the most common of all medical disorders worldwide and, while for some it represents no more than a nuisance, for others it imposes significant negative impacts on daily life and activities. IBS is a heterogeneous disorder and may well have a number of causes which may lie anywhere from the external environment to the contents of the gut lumen and from the enteric neuromuscular apparatus and the gut immune system to the central nervous system. Consequently, the paradigm of the gut-brain axis, which includes the participation of these various factors, has proven a useful model to assist clinicians and patients alike in understanding the genesis of symptoms in IBS. Now, given the widespread interest in the gut microbiome in health and disease, in general, reports of disordered enteric bacterial communities in IBS, and experimental data to indicate that components of the gut microbiota can influence brain morphology and function, as well as behavior and cognition, this concept has been extended to encompass the microbiota-gut-brain axis. The implications of this novel concept to the assessment and management of IBS will be explored in this review.

## 1. IBS—An Introduction

Irritable bowel syndrome (IBS) is now recognized to be common worldwide and to be most common among adolescent and young adult females [1,2,3]. The clinical and socioeconomic impact of IBS varies—for some it is a nuisance, for others it disrupts every aspect of their daily lives—work, school, personal and social relationships [4]. IBS is not a new disorder; what is new is its acceptance as a “real” and potentially incapacitating disorder by the health care profession and the general public alike. The era when IBS patients were dismissed as “neurotic”, self-absorbed and in need of no more than a good talking to, where they were encouraged to “straighten themselves up”, is hopefully in the past. With this greater recognition of the true personal and societal impact of IBS has come an appreciation that IBS is deserving of serious clinical and scientific study. While real progress has been made and new therapeutic targets identified, the barriers to progress in this disorder remain formidable. Foremost among these is the well documented heterogeneity of the IBS phenotype; it seems likely that IBS is not a single entity but rather a spectrum manifest by a variety of clinical presentations with a number of underlying causes and a range of aggravating factors. Progress has also been hampered by the absence of a validated and uniformly applicable biomarker for IBS—no surprise, surely given its intrinsic heterogeneity. Consequently, IBS continues to be defined by its dominant symptoms. Here real progress has been made through the ongoing efforts of the Rome process—now in its 4th iteration [5]. The deliberations of the Rome committees result in the promulgation of symptom-based criteria for the diagnosis of various functional gastrointestinal disorders, including IBS. These are developed through an evidence-based and expert-informed consensus process and subsequently subjected to validation in the field.

## 2. The Gut-Brain Axis—The Quintessential IBS paradigm

The concept of the gut-brain axis, a bidirectional channel of communication between the “big brain” in the cranium and the “little brain” (i.e. the enteric nervous system) was introduced to describe the central and peripheral effects of gut-brain peptides such as cholecystokinin and bombesin [6] and was soon implicated in disorders such as anorexia nervosa [7]. As the morphology and function of the enteric nervous system (ENS) came to be fully described, it became clear that the ENS and central nervous system (CNS) shared many features other than certain biologically active peptides and the intricacies of the interactions between these nervous systems came to be appreciated. The formal description of the gut-brain axis and its adoption as a paradigm to understand the various manifestations of IBS has since served as a valuable model, not only to the clinical investigator in his or her search for insights into the disorder, but also to the sufferer to whom it provides an approachable and personally meaningful explanation for the development ant exacerbation of his or her symptoms [8].

This is not a new idea; the ability of the stressed brain to disturb colonic function was elegantly described by Almy and his colleagues 70 years ago [9]. Since then, virtually every gastrointestinal organ and every gut function has been shown to be vulnerable to the effects of stress [10,11,12,13,14,15,16]. Not surprisingly, given its recognition as a common exacerbant of symptoms in IBS, a series of clinical studies went on to demonstrate the particular sensitivity of IBS subjects to the gastrointestinal motor, sensory and secretory effects of both acute and chronic stressors [11,14,15,16,17]. In addition, in IBS, brain function may be disordered as evidenced by the frequency of co-morbid depression and anxiety; disorders that may also impact on gut function and, at the very least, complicate the study of any bodily function in relation to IBS per se.

More recently, the advent of a number of imaging modalities and sensing systems has facilitated the exploration of the other side of the coin—the perception, by the brain of gut function and dysfunction. These studies have to a variable extent documented aberrant brain responses to both physiological and pathological visceral events in IBS [18,19,20,21].

Interactions between brain and gut extend well beyond the bounds of the stressed, anxious or depressed gut and should also encompass situations where the brain, the gut and their linkage, through the autonomic nervous system, are affected by the same pathologic process, as in Parkinson’s disease (PD) as well as those instances where neurologic symptoms are a consequence of a primarily gastrointestinal pathology, as in the malabsorption syndromes [22].

## 3. The Microbiota-Gut-Brain Axis

Most recently, connections between the gut and the brain have been extended to include a new participant; the microbiota leading to the concept of the microbiota-gut-brain axis. Indeed, tantalizing evidence has emerged from animal models to suggest that bacteria resident in the gut could impact on the “big brain” and even contribute to neurological and neuropsychiatric disease. Accordingly, the microbiome has emerged as a potential therapeutic target in disorders as diverse as IBS, Parkinson’s disease and depression [23,24]. While many other neuropsychiatric disorders including Alzheimer’s disease, amyotrophic lateral sclerosis, autism, stroke and drug addiction have been linked, in one way or another, to the microbiome [25], this review will briefly focus on IBS.

**Figure 1:**
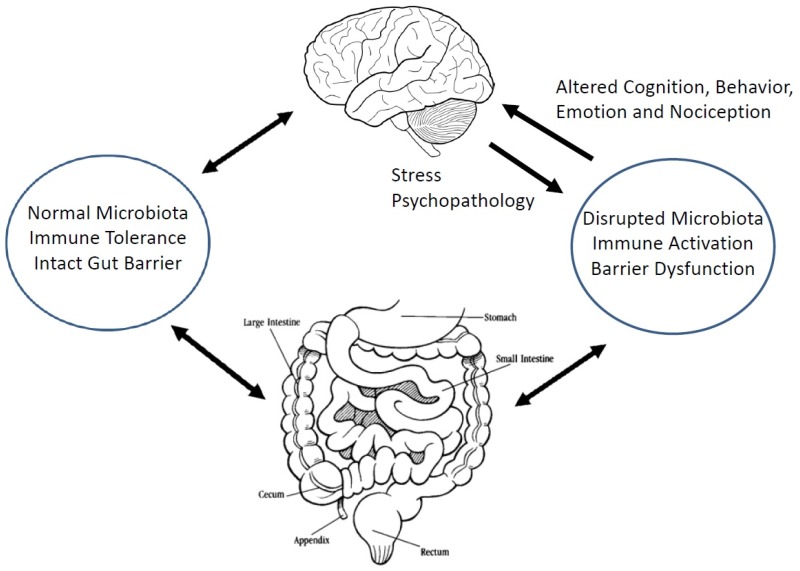
A schematic representation of the microbiota-gut-brain axis. An abnormal microbiota associated with a disrupted gut barrier and the activation of the mucosal immune system leads to the release of inflammatory mediators and other neuroactive molecules into the systemic circulation from where they reach the brain and result in changes in cognition and behavior. Alternately, central stimuli, such as stress, can disrupt mucosal immunity, the gut microbiota and gut barrier function and lead to gut dysfunction. Note: bidirectional nature of the relationships between the gut, the microbiota and the brain.

Parenthetically, it must be noted that the concept of a microbiota-gut-brain axis (and, indeed, a microbiota-gut-liver-brain axis) is far from new and was well described over 50 years ago in seminal studies on the pathogenesis of hepatic encephalopathy (HE) which identified the centrality of the gut microbiota to the genesis of this syndrome and described the beneficial impact of an intervention that modified gut bacterial population; a poorly absorbed antibiotic, on the symptoms of this syndrome [26,27]. Indeed, the model developed to explain the pathophysiology of HE; namely, the convergence of small intestinal bacterial overgrowth (SIBO) and/or an abnormal microbiota, impaired gut barrier function, a pro-inflammatory state and the appearance in the systemic circulation of neuro-active molecules generated by bacterial metabolism has become virtually ubiquitous in relation to most disorders where a role for the microbiota-gut-brain axis has been invoked [28] (Figure 1).

## 4. The Microbiota-Gut-Brain Axis and IBS

That the microbiota might be a factor in IBS [29,30] was first suggested by the observation that IBS could develop de novo in the aftermath of acute enteric bacterial, viral and parasitic infections [31]. More recently, modern sequencing technology has been applied to the study of the fecal and colonic microbiota in IBS, in general, and relationships between a variety of clinical and demographic parameters and the microbiota investigated. While data remains limited and not always consistent, it is evident that IBS patients have an altered fecal microbiota relative to healthy individuals [32]. Currently available data are fraught with challenges in interpretation—small study populations, variations in patient selection and methodology not to mind the failure to account for such confounders as diet, therapy, co-morbid psychopathology and symptom severity [33]. Nonetheless, some overall patterns have emerged: the fecal and colonic mucosal microbiota are different in IBS [32] and the fecal microbiota may not only predict severity [34] but also responsiveness to one common intervention—the low fermentable oligosaccharide, disaccharide, monosaccharide and polyol (FODMAP) diet [35]. We can look forward to more, larger and more detailed studies in this area, studies that may yield diagnostic signatures identifying new, coherent, IBS sub-populations and that are predictive of a range of therapeutic possibilities.

**Figure 2:**
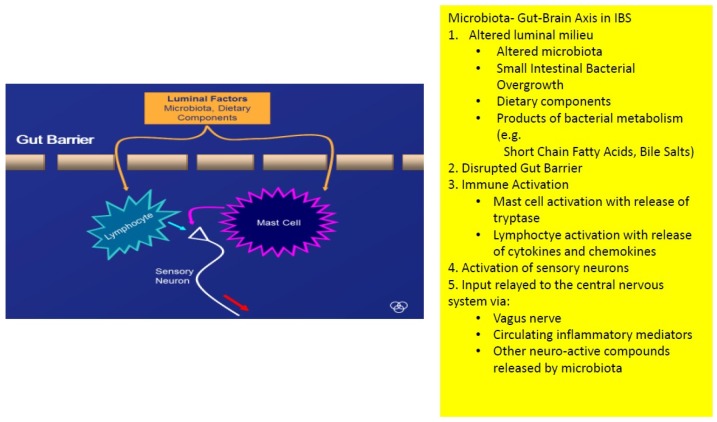
How the microbiota-gut-brain axis might operate in irritable bowel syndrome.

Small intestinal bacteria might also play a role: though its precise prevalence and role in IBS remain uncertain, evidence has been presented to associate small intestinal bacterial overgrowth with IBS [36,37]. Whatever the role of SIBO in IBS, in general, it is evident that, at the very least, SIBO can generate symptoms that are very similar to those that typify IBS. The missing link here is a precise delineation of the small intestinal microbiota in IBS; a development that will get us beyond the limitations of current methodologies such as jejunal aspiration or breath tests. Other roles for a disturbed microbiome or an aberrant host response to the microbiome, relevant to IBS, could also be invoked—the generation of intraluminal molecules with biological effects on gut function, impaired gut barrier function, immune activation and, of course communication with the central nervous system [38]. Each of these phenomena has been demonstrated in one or another IBS population and can certainly be linked to the genesis of IBS symptomatology (Figure 2) [38].

The final and, perhaps, most clinically sound evidence for the role for gut bacteria in IBS comes from clinical trials relating to two interventions that modulate the microbiome, antibiotics and probiotics, in IBS. The poorly absorbed antibiotic rifaximin [39] and probiotics, in general [40], have been shown to ameliorate the cardinal symptoms of IBS. Exactly how either intervention achieves this effect is unclear; for probiotics, in particular, effects on gut-brain signaling, motility, visceral sensation, the gut barrier and the mucosal and systemic inflammatory responses have been demonstrated in a variety of animal models and any one or a combination of these effects could be relevant [41]. The rifaximin story may be more complicated than it would appear at first sight—its effects in IBS may not be dependent on the eradication of SIBO [42] or on the modulation of bacterial populations [43]. There are many unanswered questions.

## 5. Microbiota-Gut-Brain Communications in IBS

One of the aforementioned confounders that complicates the interpretation of IBS studies deserves special mention—co-morbid psychopathology. Experimental models have revealed the ability of modifications of the microbiota to influence behavior, cognition, mood and related neurotransmitter function in experimental animals [44,45,46,47] and have also presented us with some clues as to how the microbiota and the brain might communicate. Given the pro-inflammatory phenotype associated with depression, the aforementioned pro-inflammatory effects of a disrupted microbiome and the anti-inflammatory actions of some probiotics may be relevant here also; experimental evidence supports this concept [48]. Other routes of influence, including the vagus nerve and microbial metabolites, have also been invoked [46] based on studies in animal models.

At this moment, and at every level, data in humans on the operation of this axis in IBS is scanty (related in large part to the challenges that any interrogation of the microbiota-gut-brain axis presents). The limited data that is available is encouraging and suggests that, not only that the axis is operative, but also that its modulation may be therapeutically beneficial [49,50]. These are early days but the future is promising.

## 6. Conclusions

It should come as no surprise, given advances in techniques to study the microbiota coupled with exciting data from animal models, that the paradigm of the microbiota-gut-brain axis has been proposed as relevant to IBS. To many it will be seen as a logical extension of that model which has become so central to our understanding of IBS—the gut-brain axis. The possibility that a disturbed microbiome or an aberrant host-response to that same microbiome might be relevant to IBS and might impact on the central nervous system is now being seriously contemplated and investigated and has the potential to open new diagnostic and therapeutic vistas on this challenging disorder.
